# Treatment of Exposed Pulps

**Published:** 1870-08

**Authors:** I. Knapp


### DENTAL REGISTER.

VOL. XXIV.] AUGUST, 1870. [No. 8.

### Original Communications.

### TREATMENT OF EXPOSED PULPS.

I. KNAPP, D. D. S.

Read before the Indiana State Dental Association, June 28, 1870.

With my present views the topic given me would require but a few short sentences for its discussion. I hope, therefore, in the brief paper I present to you to-day I may be pardoned a hasty glance at the past, and for introducing some matter, which may not be considered entirely germane to the subject.

If I succeed in awakening a deeper interest in our profession, or in suggesting an inquiry in any mind, which may lead to better results, to a wider sphere, and a clearer conception of dental science than have been obtained I shall be fully satisfied.

The treatment of exposed pulps has been for many years the gravest problem of dental surgery. Previous to the advent of modern dentistry the idea of saving the pulp seems not to have been entertained; but the Dentist feeling the reproach of being compelled to apply the forceps to every aching tooth, which he claimed it was his mission to preserve, began to enquire seriously for a better way. Ever watchful of facts; ever observant of the operations of natural laws, he often found the breach which the destructive agents had wrought in the solid walls which surround this most delicate and sensitive organ, had been repaired by an inner wall, built up behind a protecting mass of decayed dentine.

Obeying the dictates of the great teacher, nature, he reasoned: "If I can allay all irritation and hermetically shut out all external influences, this sensitive little organ will be quite comfortable; or if not entirely satisfied with its new relations it will be only sufficiently alarmed to prompt it to gather new material, and speedily repair the breach in its protecting walls." Prompt to act upon his conclusions, with his creasote and morphine, and many other agents, he combated, and generally successfully, both pain and irritation, Then he prepared his cap, his shield of protection; first of precious and then of base metals; and of bone, horn, quill, and rubber. Upon one and another of these materials he built up a secure and beautiful filling; and felt that he had done a good thing. Nay, so sure were the evidences of success that he often cried out in exultation, Eureka! Eureka! But alas, how soon despair triumphed over exultation, and reigned supreme.

He had not shut out that, to the pulp, most destructive of all agents, great and sudden alternations of temperatures. His cap did not fit, nor mould itself to the recess. It sometimes pressed upon it, but often left an intervening space, into which the pulp swelled, or sent a morbid growth, and fretted itself against the rough edges of the aperture.

Then followed just a little tenderness, a little soreness, a slight elongation of the tooth, and then increased arterial action, pain, distended capillaries, infiltration, heat, tumefaction, agony, death; a puss secreting sack and an abscess.

> "It will but skin and film the ulcerous place,
> While rank corruption marring all within
> Infects unseen."

Abandoning a practice, based, undoubtedly, upon a true theory:

> "That 'kept' the word of promise to our ear
> But 'broke' it to our hope."

With unabated determination to succeed the Dentist sought for other ways to save teeth with exposed pulps. Often finding the pulp dead he removed it, cleansed the canals and filled them and the decayed cavity. Watching these teeth for years, he found they retained a feeble vitality, but sufficient to retain them in their sockets answering all the purposes of sound teeth.

If a tooth, with a dead pulp, can be saved and made useful (while one with a live, exposed pulp can not) then why not put the tooth in a condition to be saved? Why not kill to make alive?

The true Dentist is ever a conservative in theory, for he seeks to preserve, to retain, and save. But to save more effectually, he is and must ever be progressive in practice. Often led by an *ignus fatius* he is ready to follow any light which seems to float over solid ground.

Hence for long years the fatal arsenic has done its deadly work. Tens of thousands of living, throbbing pulps have succumbed to this powerful poison; but

> "Death came quick and pangless."

And tens of thousands of teeth that would have been doomed to the forceps are now performing all the functions of sound organs. A practice which in skillful, experienced hands, saved, for a term of years, so large a per centage of teeth with exposed pulps, can not be condemned, for no better was known; but the process is laborious, taxing the utmost energy and skill of the operator; and laying a heavy burden upon the time and purse of the patient.

Thus, gentlemen, in general terms, I have given you my experience in the treatment of exposed pulps; and I presume it is essentially the experience of every Dentist, who has been in practice fifteen or twenty years.

Your committee ask, "What are the indications for destroying the pulp?"

Had the question been asked less than a brief year ago I should have answered, unhesitatingly, the simple fact of its exposure, if you wish to save the tooth.

But I rejoice to quote one most eminent among us that "we have received new illuminations." Our course is onward, and must be until we reach a perfected science, which is law.

Ox. chl. zinc., for many years a comparatively useless article upon the Dentist's case, has suddenly become a life-preserver, a true artificial bone. Time must test this new practice, as it has others, which have preceded it. Its present fair promise may end in disappointment. I have seen no infallible evidence of its success. I have not as yet removed any filling to see if new dentine had formed over the pulp, or whether the pulp itself was alive. I have only the negative evidence of perfect freedom from any abnormal symptoms.

Assuming, therefore, what I believe that exposed pulps can be successfully treated with ox. chl. zinc, I shall state, negatively, the conditions under which they should not be destroyed:

1st, Any accidental slight exposure.

2d. Any small exposure, which has given notice of its presence by slight pain; only *on contact* with irritating substances.

3d. Large exposures which have been so protected with thick layers of decayed dentine that the pulp has escaped all irritation.

Nearly all these cases may be prepared; touched with some escharotic, oxychloride of zinc., applied and filled, permanently, at once.

4th. All cases where the pulp, dentine, and investing membranes are in pathological condition when the normal condition can be permanently restored by treatment.

Of the four classes named I have filled twenty-six teeth of which I have some record. I have filled, perhaps, ten or twelve more of which I have none. One of the twenty-six coming under the head of the fourth class, I filled against my judgment, as the patient would submit to no treatment. The tooth was removed the second day after filling. Six or eight of the whole number are still on trial, filled only with ox. chl. zinc ; but they are all apparently doing well.

In answering affirmatively the question of your committee "What are the indications for destroying the pulp?" I am happy to say they are few.

1st. When you are satisfied that inflammation has reached that stage when the membranes and pulp begin to soften and break down (unless you wish to save a part of the organ alive) you will save yourself trouble, and your patient much suffering by the prompt use of arsenic.

This difficulty will be met, I apprehend, in the cachectic and strumous diathesis, and in persons of low vitality and feeble nutrition, especially if they be pregnant and nursing women.

The demands of the child during utero-gestation and nursing, upon the mother are often greater than her feeble powers can supply. But it seems to be a law of nature that the new shall grow at the expense of the old; that the decaying vitality of the one shall support the growing organ of the other. The mother becomes pale, thin in flesh, and feeble; the child grows strong and healthy. Especially do the teeth of the mother suffer from want of nutrition, and may it not be a question whether they are not deprived of existing bone material by the great demands of the fœtus? May we not find in this want of nutrition an explanation of that painful train of pathological conditions from which pregnant women often suffer so intensely, rather than in that high sounding but meaningless expression, sympathy?

What bond of sympathy can there be between organs so far removed and so different in anatomical structure, and in function?

May we not, therefore, class pregnant and nursing women of low vitality in the strumous and cachectic diathesis? With these classes of persons the chances of controlling, successfully, irritation and inflammation of the pulps and membranes are not flattering, and it would probably be good practice not to fill the cavity permanently until the lapse of considerable time.

It may be questioned whether the oxychloride of zinc has any therapeutical agency in saving pulps, if the case has been properly prepared for its application. But if the dentine is still sensitive, and the pulp not perfectly protected by coagulum, its escharotic effects will be promptly felt, and it may be relied upon, to a certain extent, to complete what should have been previously accomplished by other agents.

Ox. chl. zinc is a perfect non-conductor. It does "hermetically shut out" or protect the pulp from sudden alternations of temperature. This, by any other mode of capping, was not accomplished. Applied in a semi-fluid, creamy condition, it moulds and adapts itself perfectly to the soft tissues, without the least pressure, and completely fills the cavity; thus preventing any swelling of the pulp or morbid, spongy growth through the aperture, and also preventing any irritation of the membranes by pressure against its rough border. It is said to be an absorbent, but being an escharotic the porosity of its surface would be so promptly filled with coagulum, from first contact of lymph, that it may be questioned whether it can be relied upon for that purpose.

This agent does not appear to possess any mysterious potency; any occult, therapeutical power over exposed pulps. There is no conflict of contending forces; no triumph of a presiding ghost over opposing enemies. With innoxious, congenial substances you inclose and confine a healthy pulp within its cavity, interposing a soft elastic cushion of coagulum, and your work is done. A fractured bone, held in position by splint and bandage, re-unites the dissevered ends by its own inherent power. So the pulp and investing membranes securely protected from irritation, close up the aperture in the wall of their cells by their own inherent power of re-production. I dwell upon this point because I wish to assign to this invaluable agent its own work; that you may not seek to accomplish by its agency what can only be done successfully by other means.

The gravest problem of dental Surgery, successful treatment of exposed pulps, seems to be solved. But let no one imagine that the difficulties have been all removed. It will still often tax the skill of the most experienced, and the manipulation of the most careful to their utmost, to control the pathological conditions of the pulp and surrounding organs.

In closing then, I congratulate you, gentlemen, that you can now so promptly allay the pain and inflammation of this sensitive organ, when exposed to influences which would cause its speedy death, and can surround it with conditions so favorable to the free exercise of its normal functions that it can speedily repair all damages, and again beat against the walls of its repaired chamber, in harmonious response to the healthy pulsations of the whole system.

